# Sugar prevalence in *Aedes albopictus* differs by habitat, sex and time of day on Masig Island, Torres Strait, Australia

**DOI:** 10.1186/s13071-021-05020-w

**Published:** 2021-10-09

**Authors:** T. Swan, E. Ritmejerytė, B. Sebayang, R. Jones, G. Devine, M. Graham, F. A. Zich, K. M. Staunton, T. L. Russell, T. R. Burkot

**Affiliations:** 1 College of Public Health, Medical and Veterinary Sciences, James Cook University, Cairns, Australia; 2 Australian Institute of Tropical Health and Medicine, James Cook University, Cairns, Australia; 3 Division of Tropical Health and Medicine, James Cook University, Townsville, Australia; 4 Mosquito Control Laboratory, QIMR Berghofer Medical Research Institute, Brisbane, Australia; 5 Australian Tropical Herbarium, James Cook University, Cairns, Australia; 6 National Research Collections Australia, Commonwealth Industrial and Scientific Research Organisation (CSIRO), Canberra, Australia

**Keywords:** *Aedes albopictus*, Sugar feeding, Mosquito ecology, Fructose, Cold anthrone

## Abstract

**Background:**

Sugar feeding is a fundamental behaviour of many mosquito species. For *Aedes albopictus*, an important vector of dengue virus and chikungunya virus, little is known about its sugar-feeding behaviour, and no studies have been conducted on this in the southern hemisphere. This knowledge is pivotal for determining the potential of attractive targeted sugar baits (ATSBs) to control this important vector.

**Methods:**

The prevalence of sugar was assessed in 1808 *Ae. albopictus* from Masig Island, Torres Strait, Australia collected between 13 and 25 March 2020. Fructose presence and content in field-collected *Ae. albopictus* were quantified using the cold anthrone assay.

**Results:**

Significantly more male (35.8%) than female (28.4%) *Ae. albopictus* were sugar fed. There was a significant interaction between sex and time of day on the probability of capturing sugar-fed *Ae. albopictus*. For both sexes, fructose prevalence and content were higher in mosquitoes caught in the morning than in the afternoon. Female *Ae. albopictus* collected in the residential habitat were significantly more likely to be sugar fed than those collected in the woodland habitat.

**Conclusions:**

These findings provide baseline information about the sugar-feeding patterns of *Ae. albopictus* and provide essential information to enable an assessment of the potential of ATSBs for vector suppression and control on Masig Island, with relevance to other locations where this species occurs.

**Graphical abstract:**

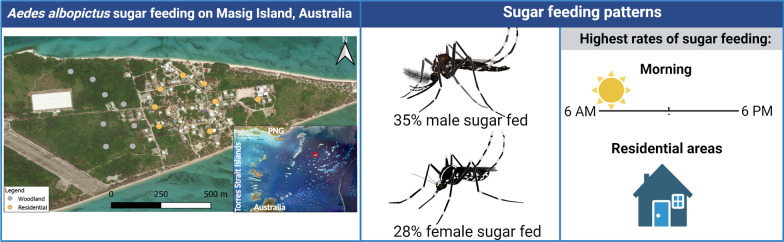

**Supplementary Information:**

The online version contains supplementary material available at 10.1186/s13071-021-05020-w.

## Background

The Asian tiger mosquito *Aedes albopictus* is highly invasive and in the past 80 years has successfully invaded every continent except Antarctica [1, 2]. The widespread dispersal of this species is concerning as it is renowned both for its nuisance biting and because it poses a risk to public health due to its ability to transmit dengue virus and chikungunya virus [3, 4]. *Aedes albopictus* is widespread within the South Pacific [5, 6] but has not yet become established on the Australian mainland, despite its broad distribution in most of the outer islands of the Torres Strait [7]. With the frequent detection of *Ae. albopictus* at points of entry across Australia [8, 9], the establishment of *Ae. albopictus* on the Australian mainland may only be a matter of time [10]. Better understanding of this mosquito’s sugar-feeding behaviour and ecology could provide insights for devising effective methods for mosquito suppression and control.

Sugar feeding is a fundamental behaviour of many species of mosquitoes [11]. Both male and female mosquitoes ingest sugar from a variety of sources, including floral and extrafloral nectar, fruit and seedpods, plant tissues, honeydew and ant regurgitate [11–14]. These sugar meals provide sustenance for basic energetic demands, such as host- and oviposition-seeking flights [11, 15]. An understanding of the sugar-feeding behaviour of *Ae. albopictus* is essential for developing attractive targeted sugar baits (ATSBs), which typically contain flower-derived attractants and sugars mixed with oral toxins [16]. The success of ATSBs is contingent on how well they can compete with naturally available sugar sources [17] and the frequency with which mosquitoes take sugar meals. Deployments of ATSBs in small-scale field trials in Florida and Israel demonstrated substantial reductions in populations of *Ae. albopictus* [18–20].

Despite the importance of sugar feeding for mosquito survivorship, and the demonstrated public health threat of *Ae. albopictus*, very little is known about the sugar-feeding behaviour of this species in nature. To our knowledge, only six field studies have investigated this behaviour, and reported that the percentage of sugar-fed individuals was moderate (defined as between 25 and 50%) to high for both males (range 48.0–67.6%) and females (range 41.8–61.5%). Additionally, these studies indicated that sugar sources, season, habitat, time of day and environmental conditions may be important in influencing sugar feeding [21–26]. For one of these factors, time of day, a consistently higher percentage of *Ae. albopictus* sugar fed in the morning than in the afternoon in a study carried out in Japan [24]. For another factor, habitat, the number of sugar-fed mosquitoes captured in a garden and wasteland site varied according to season: in summer, more sugar-fed *Ae. albopictus* were captured in the garden site compared with the wasteland site, but in autumn the opposite was true [23]. These studies suggest that the sugar-feeding patterns of *Ae. albopictus* are complex and potentially involve multiple interacting factors (e.g. season, time of day and habitat).

Chemical tests of gut contents can provide evidence of recent sugar feeding in an insect. The most popular method, the cold anthrone assay [27–29], for detecting fructose, has been successfully employed for over 40 years [30]. Primarily used as a qualitative assay (presence/absence of fructose), the use of analytical instruments (e.g. microplate readers) additionally allows for precise quantitative measures of fructose. If insects are not tested on the day they are killed, they need to be stored to prevent the enzymatic degradation of fructose [29]. Various storage methods have been used, but the reliability of these methods to maintain a stable fructose content has not been compared.

In the present study, we first compared different storage methods for the maintenance of a stable fructose content in *Ae. aegypti* measured using the cold anthrone assay. Informed by the results of these experiments, we then investigated the sugar-feeding behaviour of *Ae. albopictus* by habitat, sex, time and flower presence (and the interactions between these factors) on an offshore Australian island.

## Methods

### Laboratory experiments

#### Mosquito rearing conditions

The *Ae. aegypti* (F_4_, *w*Mel *Wolbachia*-infected) used in these experiments were sourced from eggs collected from oviposition traps deployed in Cairns in 2019 and maintained in a colony using standard laboratory rearing protocols [31]. Egg strips were hatched in two 3.4-L white buckets, each bucket containing fresh baker’s yeast (0.53 g/2 L of tap water). After 24 h, larvae were transferred to buckets containing approximately 2 L of tap water (ca. 120 larvae/bucket). Larvae were maintained on a diet of TetraMin Tropical Tablets (Tetra, Germany) ad libitum. For their use in experiments, pupae were sexed and sequentially transferred to labelled cups (20 pupae per cup).

The *Aedes albopictus* used to establish the baseline fructose-positive cutoff levels for the field studies were sourced from Hammond Island (Torres Strait, Australia) in 2016, and subsequently maintained under quarantine at the Mosquito Control Laboratory, QIMR Berghofer Medical Research Institute. Egg strips were hatched as described above and larvae were reared at a density of 400 individuals in 3 L of rainwater. Larvae were provided ground TetraMin Tropical Flakes (Tetra) ad libitum. Pupae were transferred to standard rearing cages (30 × 30 × 30 cm; Bugdorm, Taiwan) for emergence. Eclosed adults were not provided access to sugar and were killed by CO_2_ asphyxiation after 24 h. Mosquitoes of both species were maintained at 28 °C and 70% relative humidity under a 12:12-h photoperiod.

#### The effect of storage on the stability of fructose for detection by the cold anthrone assay

Laboratory experiments were used to evaluate storage conditions for the later detection of fructose in fructose-fed *Ae. aegypti* with the cold anthrone assay. Two-day-old female *Ae. aegypti* were provided with 50% fructose solution [d-(−)-fructose ≥ 99%; Merck, Australia] ad libitum for 24 h, after which they were knocked down with insecticide (Mortein Fast Knockdown Multi Insect Killer aerosol; 1.0 g esbiothrin/kg, 0.3 g permethrin/kg, 0.2 g imiprothrin/kg). The abdomen of each mosquito was visually assessed for the presence of fructose (i.e. clear liquid); only mosquitoes with clear liquid present in the abdomen were used for the subsequent experiments. Whole or crushed mosquitoes were individually stored in 1.7-mL tubes. The sugar-fed *Ae. aegypti* specimens were stored under the following conditions: (i) intact at − 20 °C (frozen), (ii) intact in 80% ethanol (EtOH) at 4 °C, (iii) heat fixed intact at 100 °C for 60 min and then stored at room temperature; (iv) crushed in 80% EtOH, and thereafter stored at 4 °C. The forceps used to crush mosquitoes were thoroughly cleaned with 80% EtOH and dried with a paper towel after each *Ae. aegypti* sample had been handled. The samples were stored for either 7, 14 or 21 days. For the EtOH treatments, on the day of testing, the EtOH in each tube was first evaporated at 100 °C for 60 min before screening the mosquitoes for the presence of fructose. For every treatment, the cold anthrone assay was used to measure the fructose content of eight female mosquitoes at 7, 14 and 21 days of storage.

#### Cold anthrone assay

The fructose content of mosquitoes was quantified using the cold anthrone assay [27] with modifications [21]. In brief, mosquitoes were homogenised using a TissueLyser II (Qiagen, USA) at 30 r.p.m. for 2 min with 50 μL of 2% sodium sulphate solution and glass beads (3 mm; Merck). Fructose was extracted by adding 375 μL of chloroform:methanol (1:2) solution, vortexing briefly and centrifuging for 15 min at 200 *g*. To quantify fructose, 10 μL of extract (or standards) was transferred to duplicate wells of a 96-well microplate and mixed with 240 μL of anthrone solution (containing 67.9 μL distilled water, 172.1 μL sulphuric acid, and 0.339 mg anthrone per sample). The plates were covered and incubated in the dark at room temperature for 90 min and absorbance was measured at 630 nm using a microplate reader (POLARstar Omega; BMG Labtech, Mornington, Australia). Standards were chosen to cover the range of the analyte, i.e. 0, 0.078, 0.156, 0.3125 and 0.625 μg/µL of D-(−)-fructose (≥ 99%; Merck) in 25% EtOH, produced once by serial dilution and stored at − 30 °C. The laboratory controls were 2-day-old female *Ae. aegypti* fed 50% fructose (positive controls) and 2-day-old female sugar-starved *Ae. aegypti* (negative controls).

#### Determination of fructose content in mosquitoes

Fructose content was calculated by subtracting the absorbance value of the blank from the sample absorbance and dividing the result by the slope of the standard curve to calculate the fructose concentration of the extract (micrograms per microlitre). The concentration was then multiplied by 425 μL (total volume of extract) to calculate the fructose content (micrograms) of the whole mosquito. The mean of the two experimental replicates of each sample was used in analyses, except when the absorbance between replicates was discordant (> 25% absorbance difference), in which case the sample was excluded from the analysis.

#### Determination of baseline fructose levels in *Ae. albopictus*

To accurately determine the sugar content of field-collected *Ae. albopictus*, laboratory-reared (96 males and 94 females), sugar-starved *Ae. albopictus* were used to establish sex-specific baseline fructose levels. Mosquitoes were reared as described above and stored intact by heat fixing at 100 °C for 60 min, followed by sugar analysis by the cold anthrone assay. Hereafter, the term ‘sugar-fed’ in relation to field-collected *Ae. albopictus* refers to a sugar content greater than the sex-specific average (+ 2 SD) of that quantitated in these laboratory-reared *Ae. albopictus*.

### Field study

#### Study site

**Fig. 1:**
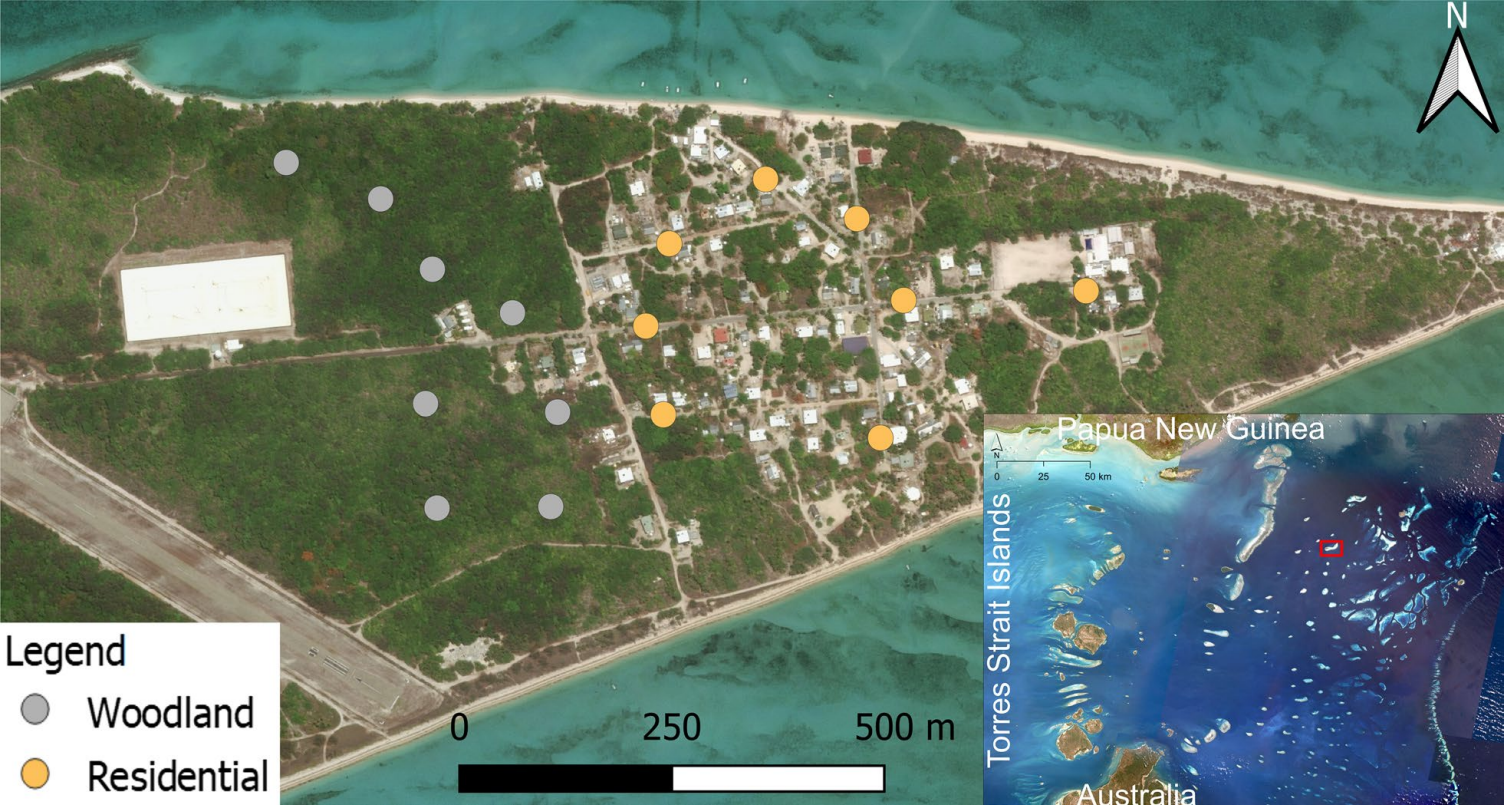
Woodland and residential stations on Masig Island, Torres Strait, Queensland, Australia where the field experiments were carried out. Note that the stations were at least 100 m apart (see Additional file 1: Table S1 for coordinates of each station and distance to either the front or back door of the nearest inhabited dwelling).* Inset* The satellite imagery shows northern Australia, the Torres Strait Islands and southern Papua New Guinea. The* red rectangle* indicates the location of Masig Island. The map was produced in QGIS with the World Geodetic System 1984 projection and the World Imagery (2020) layer. *Inset* The satellite imagery was modified from the Torres Strait Clear Sky Landsat (https://eatlas.org.au/data/uuid/71c8380e-4cdc-4544-98b6-8a5c328930ad)

Located in the tropics, the Torres Strait region experiences distinct dry (May–October) and wet (November–April) seasons. The temperature varies marginally throughout the year, with average minimum and maximum temperatures of 24.4 °C and 30.9 °C in the dry season and 25.8 °C and 32.2 °C in the wet season, respectively [32]. The estimated annual rainfall is 1452 mm, of which the vast majority falls in the wet season [32]. The study site was on Masig Island, a small coral cay (2.7 km long, and 800 m at its widest point), located in the Central Islands group of the Torres Strait (Fig. 1). The island has a population of ca. 270 people [33]. See Swan et al. [34] for further information about the study site.

**Fig. 2:**
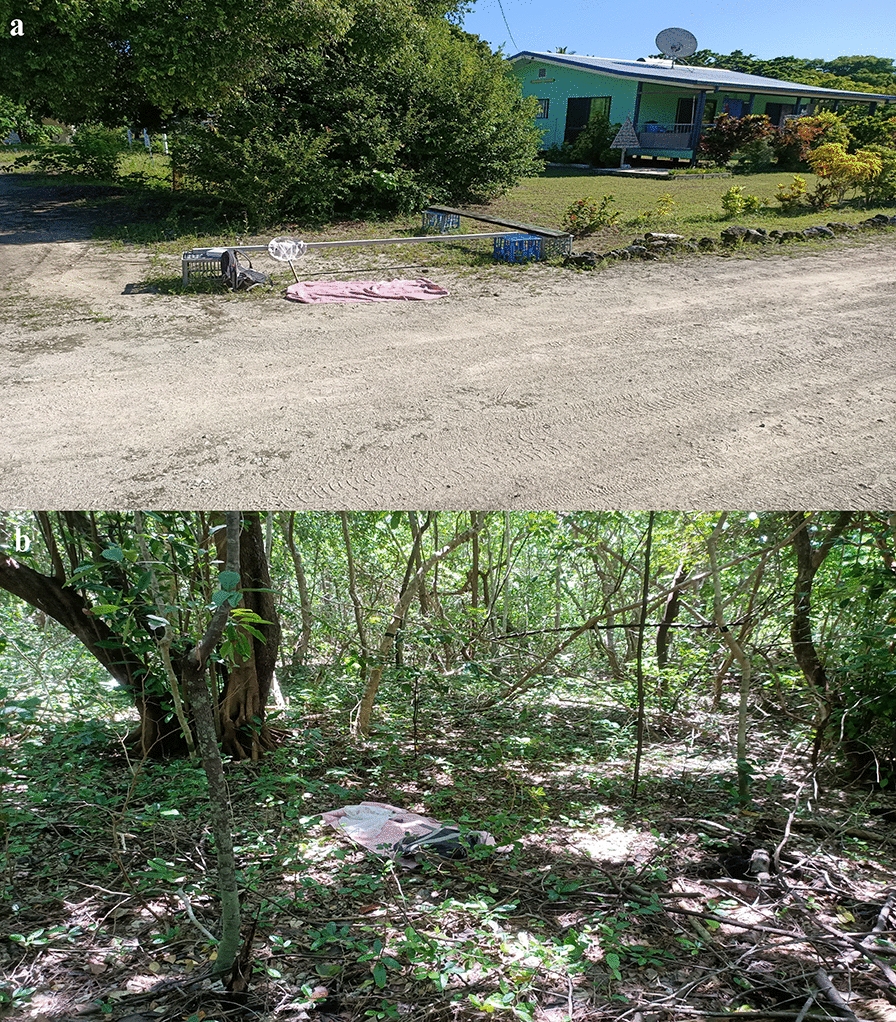
Mosquito sampling at stations in **a** residential and **b** woodland habitats on Masig Island, Torres Strait, Queensland, Australia

*Aedes albopictus* were sampled at specific stations within two habitats. ‘Habitat’ refers to the defined habitat type (woodland or residential) where the mosquito sampling was carried out (Figs. 1, 2). ‘Station’ refers to the exact location within a habitat where mosquito sampling was carried out. The inclusion criterion for woodland stations required them to be within the characterised regional ecosystem 3.2.6b: *Casuarina equisetifolia*-dominated woodland to open forest, occasionally with a sub-canopy of vine thicket species [35]. The inclusion criterion for residential stations required them to be outside this regional ecosystem and up to 5 m from the boundary of an inhabited property. Houses in the residential habitat were typically low-density, single-storey dwellings. Sixteen stations were randomly selected: eight in the woodland habitat and eight in the residential habitat. Each station was at least 100 m from the other stations. For each station, the distance to either the front or back door of the nearest inhabited dwelling and the coordinates are provided in Additional file 1: Table S1. All mosquito sampling was performed by the same collector. The order of visitation for both habitat and station was randomised before each sampling period. A Microsoft Excel random number generator between 1 and 2 was run on Woodland (= 1) and Residential (= 2) to determine which habitat would be sampled first. Another random number generator between 1 and 8 was then run to determine the order of visitation for stations within each habitat.

#### Study period

*Aedes albopictus* were collected during the wet season from 13 to 25 March 2020. Weather data were obtained from the nearest Bureau of Meteorology weather observation station on Poruma Island (~ 50 km from Masig Island). Total rainfall for this period was 189 mm [32]. The minimum and maximum temperatures were 25.4 °C and 31.8 °C, respectively [32].

#### Mosquito sampling and habitat types

Mosquitoes were sampled twice daily, from 0600 to 1000 hours and 1500–1900 hours. A 38-cm-diameter sweep net (Australian Entomological Supplies, NSW, Australia) treated with insecticide (Mortein Fast Knockdown Multi Insect Killer aerosol; 1.0 g esbiothrin/kg, 0.3 g permethrin/kg, 0.2 g imiprothrin/kg) was used to sample for 10 min or until ten *Ae. albopictus* had been captured per station (we attempted to capture five males and five females per station) at each time point. The captured insects were removed from the net and examined with a Carson TV-15, TriView magnifier (×15 magnification) for sex and species identification [36] before transferring each mosquito into a labelled 1.7-mL microcentrifuge tube. Mosquitoes were held in microcentrifuge tubes for no longer than 60 min prior to drying them at 100 °C for 60 min (with the lid open) using either a Genius Dry Bath Incubator or a Ratek Dry Block Heater. Dried mosquitoes were then stored at room temperature in ziplock bags containing silica beads.

#### Plant census

A presence-absence plant census was conducted once along a 3-m × 30-m transect at each mosquito sampling station during the study period. Potential sugar sources, i.e. plants with blooming flowers and/or with fleshy fruits (whole or damaged), were recorded at each station. Extrafloral nectaries, specialised nectar-secreting plant glands, are highly diverse in location, form and size [37]. For these reasons, their presence in recorded plant species was confirmed off-site by an expert (Australian Tropical Herbarium, James Cook University). Plants were photographed and identified either in the field or later in accordance with Nelder et al. [35], Smith [38], and Stanton et al. [39].

### Statistical analysis

#### Laboratory experiments

To investigate differences in fructose content between each preservation method, a generalised linear model (GLM) was fitted using R Studio [40]. Initial models tested all main effects and the interaction between the parameters Day and Treatment, with the log-transformed (to stabilize the variance) fructose content as the response variable. The interaction between Day and Treatment was not significant, therefore the simplified model with the form: log fructose content ~ Day + Treatment was used. The predictor variables were evaluated with an analysis of deviance using the car package in R (version 3.0; [41]). Finally, Tukey post hoc comparisons to determine significant differences among the estimated marginal means (least squares means) of treatment groups were performed by using the emmeans package in R (version 1.4.6; [42]).

#### Field studies

To investigate the proportion of sugar-fed *Ae. albopictus* by sex, time of day and habitat type, a generalised linear mixed-effects model (GLMM) with a binomial distribution was fitted using the glmer function in the lme4 package (version 1.1; [43]) in R Studio [40]. The sugar-fed status of each mosquito, as determined by the cold anthrone assay, was the binary response variable. The presence or absence of blooming flowers at a station, as determined by the plant census, was included as an explanatory variable. Initial model runs tested all main effects and possible interactions between the parameters Sex, Time of day, Habitat type and Flower presence (all fixed effects), with the sugar-fed status as the response variable. Day and Station were treated as random effects in the model. Non-significant interactions were removed from the model, leaving a simplified model with the form: sugar-fed status ~ Sex × Time + Habitat + Flower presence + (1|Day) + (1|Station). To investigate the Sex: Tme of day interaction further, the data were partitioned by sex and separate female and male models were run (no interactions between the fixed effects were found in the sex-separated models). These simplified models were: sugar-fed status ~ Time + Habitat + Flower presence + (1|Day) + (1|Station). A Hosmer–Lemeshow goodness-of-fit test was calculated (hoslem.test function in the ResourceSelection package in R) (version 0.3; [44]), and indicated that there was no evidence that any model was misspecified (*P* > 0.05). To further understand the factors influencing the magnitude of sugar feeding, a linear mixed-effects model (LMM) was employed to evaluate log-transformed fructose content of sugar-fed mosquitoes, using all the fixed and random effects listed above (function lmer in the lme4 package in R). Once again, three models were run: an overall model with both male and female data, and sex-specific models. For all GLMMs and LMMs, the effect of the fixed effects were evaluated by an analysis of deviance in the car package in R (version 3.0, [41]).

## Results

### Laboratory experiments

**Fig. 3:**
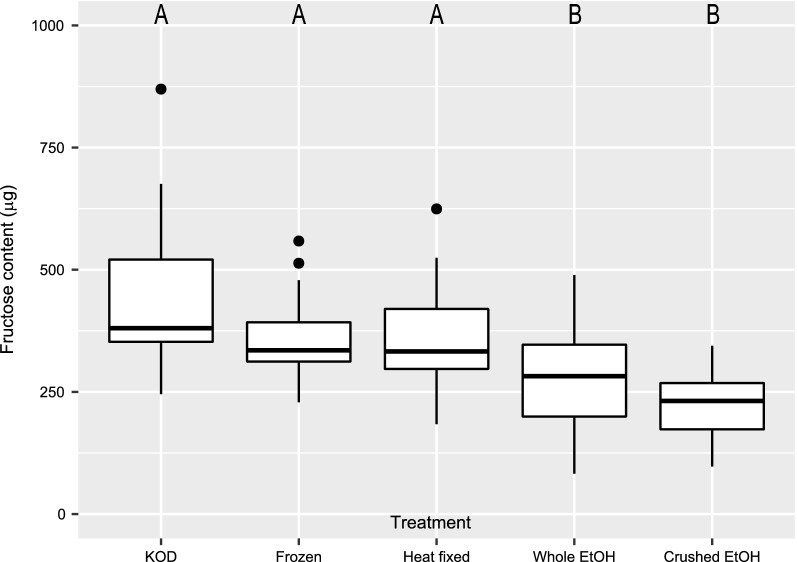
Fructose content of female *Aedes aegypti* by preservation method (*n* = 24 female *Ae. aegypti* for each treatment). Data shown for mosquitoes stored for 7, 14 and 21 days.* KOD* Female *Ae. aegypti* killed on the day of testing;* Frozen* female *Ae. aegypti* stored intact at − 20 °C;* Heat-fixed* female *Ae. aegypti* heat fixed at 100 °C for 60 min, thereafter, stored at room temperature;* Whole EtOH* female *Ae. aegypti* stored intact in 80% ethanol (EtOH), thereafter, stored at 4 °C;* Crushed EtOH* female *Ae. aegypti* crushed in 80% EtOH, thereafter, stored at 4 °C. Different letters indicate significant differences between groups (*P* < 0.05, Tukey honest significant difference)

There was a significant difference in fructose content of *Ae. aegypti* by storage method (*χ*^2^ = 59.1, *df* = 4, *P* < 0.001; Fig. 3). Fructose content of *Ae. aegypti* killed on the day of collection did not differ between the frozen (*P* = 0.45) and heat-fixed (*P* = 0.33) treatments, but was significantly higher in these treatments compared to those in which *Ae. aegypti* was stored crushed (*P* < 0.0001) or whole in 80% EtOH (*P* < 0.0001). There was no significant difference in fructose content between *Ae. aegypti* stored crushed or whole in 80% EtOH (*P* = 0.24). For each storage method, there was no significant difference in fructose content of *Ae. aegypti* after 7, 14 and 21 days in storage (*χ*^2^ = 0.37, *df* = 2, *P* = 0.83).

### Field studies

#### Field collection

Across 11 days of sampling, 1808 *Ae. albopictus* were collected, of which 1049 were females (58.0%) and 759 males (42.0%). More *Ae. albopictus* were captured in woodland (67.2%) than at residential stations (32.8%). Of these, more males (52.1%) than females (47.9%) were captured in woodland stations, but more females (78.8%) than males (21.2%) were captured in residential stations. In the morning, more female *Ae. albopictus* (56.0%) than male *Ae. albopictus* (44.0%) were captured. Likewise, in the afternoon, more female *Ae. albopictus* (59.8%) than male *Ae. albopictus* (40.2%) were captured.

#### Sex-specific baseline fructose content

The fructose contents of all sugar-starved, 1-day-old laboratory-reared male and female *Ae. albopictus* were above 0 µg. The average (+ 2 SD) fructose content in sugar-starved mosquito samples was 0.755 (+ 2.200) µg for female *Ae. albopictus* and 0.608 (+ 1.713) µg for male *Ae. albopictus*. All field-captured female *Ae. albopictus *with fructose contents greater than 2.955 µg and male *Ae. albopictus* with fructose contents greater than 2.321 µg were considered sugar fed.

#### Sugar fed status

A moderate percentage of both male (35.8%) and female (28.4%) *Ae. albopictus* were sugar fed. The percentage and fructose content of sugar-fed male and female *Ae. albopictus* by habitat, time of day and flower presence are displayed in Table 1. Among sugar-fed *Ae. albopictus*, the mean (± SEM) fructose content was 23.2 (± 1.62) μg for female and 13.7 (± 1.0) μg for male *Ae. albopictus*.

**Table 1:** Average fructose content and percentage of sugar-fed female and male *Aedes albopictus* by habitat type, time of day and flower presence

	Sugar-fed females			Sugar-fed males		
	*n* (%)	Fructose content (± SEM) (µg)	Total	*n* (%)	Fructose content (± SEM) (µg)	Total
Habitat type						
Woodland	150 (25.8)	22.8 (± 2.0)	582	233 (36.8)	13.9 (± 1.1)	633
Residential	148 (31.7)	23.6 (± 2.6)	467	39 (31.0)	12.6 (± 2.0)	126
Time of day						
Morning	165 (35.3)	23.7 (± 2.1)	467	191 (52.0)	14.9 (± 1.2)	367
Afternoon	133 (22.9)	22.5 (± 2.5)	582	81 (20.7)	10.7 (± 1.9)	392
Flower presence						
Present	189 (28.3)	22.3 (± 2.1)	668	135 (35.2)	12.7 (± 1.3)	384
Absent	109 (28.6)	24.7 (± 2.5)	381	137 (36.5)	14.6 (± 1.5)	375

Statistical tests of these differences are shown in Tables 2 and 3

#### Plant census

The identified plant species in residential and woodland habitats are presented in Additional file 2: Table S2. The average species richness (± SEM) was 8.1 (± 0.72) and 2.6 (± 0.18) in the residential and woodland habitats, respectively. Stations in the woodland habitat only contained woody plants. *Casuarina equisetifolia* (Casuarinaceae), *Guettarda speciosa* (Rubiaceae) and *Drypetes deplanchei* (Putranjivaceae) were the most frequently recorded species in woodland stations. Stations in the residential habitat were mostly dominated by herbaceous plants, and the distribution of woody plants was patchy. The most common herbaceous plants recorded in residential stations were *Tridax procumbens* (Asteraceae), *Catharanthus roseus* (Apocynaceae) and *Hibiscus rosa-sinensis* (Malvaceae). *Archontophoenix alexandrae* (Arecaceae) was the most common woody plant recorded at stations in the residential habitat. The presence of flowers was recorded at every residential station. In the woodland habitat, the presence of flowers was recorded at three of the eight stations. The presence of fleshy fruits was only recorded at three of the eight stations in the residential habitat. In contrast, fleshy fruits were present at seven out of eight stations in the woodland habitat. Extrafloral nectaries were confirmed for ten plant species. The presence of extrafloral nectaries was recorded at all eight stations in the residential habitat, but they were absent from plants at all the stations in the woodland habitat.

### The effects of measured parameters on the presence of sugar feeding

The results from the GLMMs showed that sex, time of day, habitat and the interaction between sex and time of day influenced the prevalence of sugar-fed *Ae. albopictus*, but flower presence did not (Table 2). A significantly higher percentage of male (35.8%) than female (28.4%) *Ae. albopictus* were sugar fed (*χ*^2^ = 57.5, *df* = 1, *P* < 0.001). There was a significantly higher percentage of sugar-fed *Ae. albopictus* collected in the morning (42.7%) than in the afternoon (22.0%) (*χ*^2^ = 50.0, *df* = 1, *P* < 0.001). There was a significant interaction between sex and time of day on the probability of capturing sugar-fed *Ae. albopictus* (*χ*^2^ = 15.37, *df* = 1, *P* < 0.001). GLMMs for the female- and male-specific models are shown in Table 2. A significantly higher percentage of both male *Ae. albopictus* (52.0%) (*χ*^2^ = 47.3, *df* = 1, *P* < 0.001) and female *Ae. albopictus* (35.3%) (*χ*^2^ = 15.9, *df* = 1, *P* < 0.001) collected in the morning were sugar fed compared to those collected in the afternoon (20.7% and 22.9%, respectively). A significantly higher percentage of female *Ae. albopictus* captured in the residential habitat (31.7%) were sugar fed compared to female *Ae. albopictus* captured in the woodland habitat (25.8%) (*χ*^2^ = 8.8, *df* = 1, *P* = 0.002). No significant difference between the percentage of sugar-fed male *Ae. albopictus* found in the residential (31.0%) and woodland habitats (36.8%) was found (*χ*^2^ = 0.01, *df* = 1, *P* = 0.91). The presence of flowering plants did not significantly influence the probability of sugar feeding for either male (*χ*^2^ = 0.15, *df* = 1, *P* = 0.69) or female *Ae. albopictus* (*χ*^2^ = 3.03, *df* = 1, *P* = 0.08).

**Table 2:** Direct model output from the generalised linear mixed model fitting the effects of measured parameters on the proportion of sugar-fed *Aedes albopictus*

Predictors	Odds ratio	SE	CI	*P*-value
**Female and male combined model**^a^				
(Intercept)	0.40	0.24	0.25–0.64	< 0.001***
Time (morning)	1.76	0.15	1.31–2.37	< 0.001***
Sex (male)	0.95	0.17	0.68–1.32	0.759
Habitat (woodland)	0.63	0.16	0.46–0.86	0.004**
Flowers	0.87	0.14	0.66–1.15	0.337
Time (morning) × sex (male)	2.38	0.22	1.54–3.66	< 0.001***
**Female-only model**^b^				
(Intercept)	0.55	0.31	0.30–1.01	0.054
Time (morning)	1.87	0.15	1.39–2.53	< 0.001***
Habitat (woodland)	0.48	0.25	0.30–0.78	0.003**
Flowers	0.65	0.25	0.40–1.06	0.081
**Male-only model**^c^				
(Intercept)	0.23	0.38	0.11–0.50	< 0.001***
Time (morning)	3.86	0.2	2.63–5.67	< 0.001***
Habitat (woodland)	0.97	0.27	0.58–1.63	0.913
Flowers	1.07	0.18	0.75–1.54	0.696

The data were analysed by three models, each of which was fitted to a binomial distribution with a logit link function. Parameters* in parentheses* are those compared to the reference levels.* Asterisks* indicate statistical significance

SE Standard error. CI Confidence interval

^a^Model 1: the proportion of sugar-fed female and male *Ae. albopictus*. The reference level for Sex is female, the reference level for Habitat is residential, and the reference level for Time is afternoon

^b^Model 2: the proportion of sugar-fed female *Ae. albopictus*. The reference level for Habitat is residential and the reference level for Time is afternoon

^c^Model 3: the proportion of sugar-fed male *Ae. albopictus*. The reference level for Habitat is residential and the reference level for Time is afternoon

### The effects of measured parameters on fructose content in sugar-fed Ae. albopictus

**Fig. 4:**
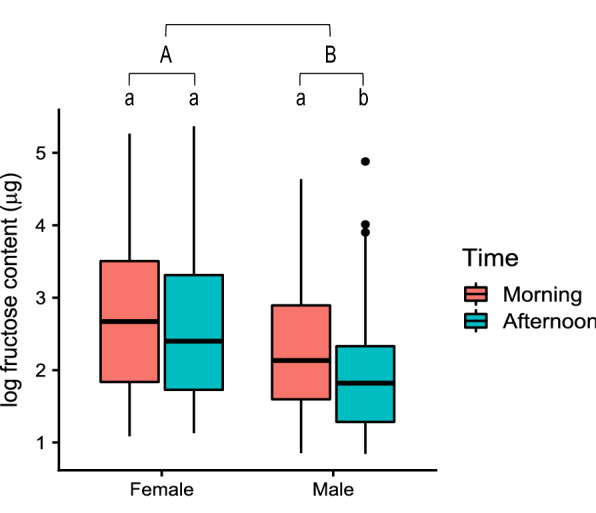
Log-transformed fructose contents of female and male *Aedes albopictus* by time of day for sugar-fed mosquitoes only. Different letters indicate significant differences between groups (*P* < 0.05, ANOVA). Capital letters indicate significant differences in fructose content between female and male *Ae. albopictus*. Lowercase letters indicate significant differences in fructose content by time of day

The results from the LMMs showed that fructose content in sugar-fed *Ae. albopictus* was predicted by sex and time of day, but not by habitat type or flower presence (Table 3). Among sugar-fed *Ae. albopictus* (*n* = 570), fructose content (mean ± SEM) was significantly higher in females (23.2 ± 1.62 μg) than in males (13.7 ± 1.0 μg) (*χ*^2^ = 34.2, *df* = 1, *P* < 0.001; Table 3; Fig. 4). For male *Ae. albopictus*, fructose content was significantly higher in individuals collected in the morning (14.9 ± 1.2 μg) than in the afternoon (10.7 ± 1.9 μg) (*χ*^2^ = 8.34, *df* = 1, *P* = 0.004; Table 3; Fig. 4). There was no significant difference in fructose content by time of day between male and female *Ae. albopictus* (*χ*^2^ = 3.06, *df* = 1, *P* = 0.08; Table 3) or among female *Ae. albopictus* (*χ*^2^ = 0.01, *df* = 1, *P* = 0.89; Table 3; Fig. 4). Among sugar-fed *Ae. albopictus*, there was no significant difference in fructose content between woodland (17.4 ± 1.1 μg) and residential (21.3 ± 2.1 μg) habitats (*χ*^2^ = 0.14, *df* = 1, *P* = 0.70; Table 3). The presence of flowers did not significantly affect the fructose content found in either female (*χ*^2^ = 1.64, *df* = 1, *P* = 0.20; Table 3) or male (*χ*^2^ = 0.03, *df* = 1, *P* = 0.86; Table 3) *Ae. albopictus*.

**Table 3:** Direct model output from the linear mixed-effects model fitting the effects of measured parameters on the log fructose content of sugar-fed *Aedes albopictus*

Predictors	Estimate	SE	CI	*P*-value
**Female and male combined model**^a^				
(Intercept)	2.65	0.16	2.33–2.97	< 0.001***
Sex (male)	− 0.50	0.09	− 0.67 to − 0.33	< 0.001***
Habitat (woodland)	− 0.05	0.14	− 0.33 to 0.22	0.709
Time (morning)	0.15	0.09	− 0.02 to 0.33	0.080
Flowers	− 0.11	0.13	− 0.37 to 0.14	0.373
**Female-only model**^b^				
(Intercept)	2.80	0.21	2.38–3.21	< 0.001***
Time (morning)	0.02	0.12	− 0.23 to 0.26	0.89
Habitat (woodland)	− 0.08	0.18	− 0.43 to 0.26	0.641
Flowers	− 0.23	0.18	− 0.58 to 0.12	0.2
**Male-only model**^c^				
(Intercept)	2.02	0.26	1.52–2.52	< 0.001***
Time (morning)	0.33	0.12	0.11–0.56	0.004**
Habitat (woodland)	− 0.07	0.22	− 0.50 to 0.36	0.745
Flowers	− 0.03	0.19	− 0.40 to 0.33	0.86

The data were analysed with three models. *Asterisks* indicate statistical significance

SE Standard error. CI Confidence interval

^a^Model 1: the log fructose content of sugar-fed female and male* Ae. albopictus*. The reference level for Sex is female, the reference level for Habitat is residential, and the reference level for Time is afternoon

^b^Model 2: the log fructose content of sugar-fed female* Ae. albopictus*. The reference level for Habitat is residential and the reference level for Time is afternoon

^a^Model 3: the log fructose content of sugar-fed male* Ae. albopictus*. The reference level for Habitat is residential and the reference level for Time is afternoon

## Discussion

A significantly higher percentage of field-collected male (35.8%) than female (28.4%) *Aedes albopictus* were sugar fed on Masig Island. This finding confirms results from previous studies on *Ae. albopictus*, although the percentages of male and female *Ae. albopictus* sugar fed in the present study are lower than previously reported (between 48.0–67.6% for male and 41.8–61.5% for female *Ae. albopictus* [21, 22, 24, 25]). Sugar prevalence in *Aedes albopictus,* as well as in *Aedes aegypti* and *Anopheles gambiae*, may be influenced by many factors, including the composition and concentration of sugar, type and availability of sugar and environmental conditions [21–23, 25, 26, 45, 46]. It is possible that the tropical climate (i.e. warm and moist weather) of Masig Island during the wet season may not be as conducive to sugar feeding as the drier climates where previous studies were conducted. In New York, the rate of *Ae. albopictus* sugar feeding increased under environmental conditions with hotter and drier weather [21]. A similar result was found in Italy, with *Ae. albopictus* sugar feeding positively correlated with temperature and negatively correlated with relative humidity [22]. It is possible that hot and dry environmental conditions dehydrate mosquitoes, which may trigger higher rates of sugar feeding. For *Ae. aegypti* in Thailand, there was over a threefold increase in sugar feeding in the dry season (16%) compared with the wet season (5%) [47]. For *Ae. aegypti* in Kenya, higher rates of sugar feeding were only observed for males in the dry season (27%) compared with the wet season (11%) [48]. Investigations of *Ae. albopictus* sugar-feeding prevalence by sex during the wet and dry seasons may illuminate differences in sugar feeding, which are possibly related to environmental conditions.

Sugar feeding by both male and female *Ae. albopictus* on Masig Island was more prevalent in individuals collected in the morning than in the afternoon. Likewise, in Japan, a consistently higher percentage of *Ae. albopictus* were sugar fed in the morning than in the afternoon [24]. Little is known about the diel sugar-feeding periodicity of *Ae. albopictus* in nature. Laboratory observations of the diel sugar-feeding periodicity of *Ae. albopictus* showed a bimodal pattern with peaks in the morning and afternoon [49]. An important consideration in interpreting these results is the time since the last sugar meal. In the present study, and in Harada et al. [24], it is uncertain whether *Ae. albopictus* collected in the morning did indeed feed during this time period. Presumably, *Ae. albopictus* captured close to the time of feeding in the field would have higher fructose contents than those that had fed hours or days previously, as has been shown under laboratory conditions [21]. The rate of digestion would also influence the fructose content, as would the time elapsed since the last sugar meal. Under laboratory conditions at 23.5 °C and 28 °C, *Ae. albopictus* fully digested (as determined by the cold anthrone assay) a 10% sucrose meal within 24 h of ingestion [21]. The concentration of sugar accessible to insects in nature likely varies considerably, and it is possible that Fikrig et al. [21] used a sugar concentration at the low end of those found in nectar (~15–70%) [50]. The rate of sugar digestion in *Ae. albopictus* under field conditions is, to our knowledge, not known, and likely varies considerably between sugar sources and environmental conditions. Mark–release–recapture experiments could be used to investigate how long a sugar meal persists in field-released sugar-fed *Ae. albopictus*; such sugar meals have been reported to persist, on average, for at least 50 h in field-released *Ae. aegypti* in Thailand [51].

The percentage of sugar-fed *Ae. albopictus* was not predicted by habitats with flowers present. Only two other studies have investigated flower abundance or presence and the proportion of sugar-fed *Ae. albopictus*, with differing results. In Israel, a higher percentage of female *Ae. albopictus* were found to be sugar fed in garden sites (68%), compared with dry wasteland sites (42%) [23]. In New York, there was no significant difference in the number of sugar-fed *Ae. albopictus* between properties with or without flowers [21]. It is difficult to compare these studies due to considerable differences in terms of the strain of mosquito examined, the ecosystem, the plant taxa present and the environmental conditions under which the studies were carried out. Additionally, in the study reported here, the quality of the flower resource, such as nectar quality and quantity, was not considered, although this is highly variable in nature [52]. Given the flight range of *Ae. albopictus* (> 200 m; [53, 54]), it is likely that *Ae. albopictus* on Masig Island opportunistically access sugar sources beyond the plants in the census. Mosquitoes obtain sugar by feeding on a wide range of sources including floral and extrafloral nectar, fruit and seedpods, plant tissues, honeydew and ant regurgitate [11–14, 55]. The results from the present study suggest that flowers are but one source of sugar, and perhaps not an important one for *Ae. albopictus* on Masig Island at the time of the survey. Furthermore, it is not entirely clear why higher rates of sugar feeding in the residential habitat was only observed for female *Ae. albopictus*. One possibility is that increased sugar feeding in the residential habitat could have been influenced by extrafloral nectar, which was only found in plant species in this habitat at the time of the plant census. This idea warrants further investigation, including the need for direct field observations of extrafloral nectar visitation by *Ae. albopictus*. Another possibility is that increased sugar feeding in the residential habitat could have been influenced by the presence of plants in the families Fabaceae and Malvaceae, which were only found in the residential habitat on Masig Island at the time of the plant census. Previous studies have identified a preference of *Ae. aegypti* for host plants in these families [48, 56]. Future studies could utilise more sophisticated molecular techniques, such as DNA barcoding or mass spectrometry, to determine the exact sources of sugar and the relative sugar-feeding frequency of *Ae.* *albopictus *in different habitat types in nature [56, 57].

Our mosquito sampling method (sweep net) captured both male and host-seeking female *Ae. albopictus*. Sugar-fed female *Ae. albopictus* consumed significantly larger sugar meals than male *Ae. albopictus*. In New York, female *Ae. albopictus* (captured with both sweep nets and aspirators) contained more sugar than male *Ae. albopictus* [21], but the opposite was true in Texas, with *Ae. aegypti* males containing more sugar than *Ae. aegypti* females captured with BG-Sentinel 2 traps, aspirators and Centers for Disease Control and Prevention resting traps [58]. These results suggest sex-specific differences between these two species in the amount of sugar consumed, although these differences are possibly the consequence of collection methods. Male mosquitoes across many species commonly swarm around humans, presumably anticipating the arrival of a female mosquito to create a mating arena [59, 60], which in turn likely leads to the rapid expenditure of sugar reserves. It is possible that the presence of a human collector (who is likely to initiate a swarm) with a sweep net greatly accelerates the rate of sugar metabolism in mosquitoes. For *Culex tarsalis* in California, individuals captured in the morning were more likely to test positive for fructose than those captured after swarming [61]. Future studies could investigate how collection methods influence both the prevalence and quantity of sugar in female and male *Ae. albopictus*, as it has been suggested that collection methods may have influenced the results of previous sugar-feeding studies across *Aedes* species [21, 58].

### The effect of storage on the stability of fructose for detection by the cold anthrone assay

In previous field investigations, *Ae. aegypti* were stored after heat fixing for later sugar detection with the cold anthrone assay [47, 62]. To our knowledge, no published study has investigated the reliability of this storage method in maintaining a stable fructose content relative to other commonly used insect storage methods. Our results demonstrate that heat fixing *Ae. aegypti* is as reliable as freezing or killing insects on the day of sugar testing. Conversely, *Ae. aegypti* stored whole or crushed in 80% EtOH had significantly lower fructose contents than *Ae. aegypti* subjected to the other storage methods tested. Enzymes responsible for breaking down fructose were likely still active in samples of *Ae. aegypti* stored crushed and whole in 80% EtOH at 4 °C. Previous research found that trehalose (stored sugar) measurements in parasitoid wasps stored whole in 70% EtOH were significantly lower than those in parasitoids either crushed in 70% EtOH or frozen at − 20 °C [63]. van Handel [29] cautions that, when storing insects for the measurement of sugars, the insects should be stored frozen at − 20 °C or heat fixed at > 90 °C, to prevent the enzymatic degradation of the sugars. Surprisingly, a few recent insect sugar studies have stored specimens in EtOH [64, 65]. Our findings corroborate those of Phillips [63], and we conclude that the storage of *Ae. aegypti* (and likely other insects) in EtOH is unreliable for the detection of fructose with the cold anthrone assay. We recommend either heat fixing or freezing specimens as reliable storage methods. When working in remote locations, the ease of transporting heat-fixed specimens is a significant advantage over the requirement for a cold chain for frozen specimens.

## Conclusions

Our results provide a basic understanding of, and insights into, factors which may influence the sugar-feeding patterns of *Ae. albopictus*. We found that a moderate percentage of both male (35.8%) and female (28.4%) *Ae. albopictus* were sugar fed. For both sexes, the prevalence of sugar feeding and fructose content were highest for individuals collected in the morning compared to those collected in the afternoon. Lastly, female *Ae. albopictus* collected in the residential habitat were significantly more likely to be sugar fed than those collected in the woodland habitat. Our results provide a foundation for future studies investigating the potential deployment of ATSBs on Masig Island or in other tropical locations where this species occurs.

## Supplementary Information

**Additional file 1: Table S1.** Coordinates for each station and distance to either the front or back door of the nearest inhabited dwelling.

**Additional file 2: Table S2.** Identified plant species at stations in woodland and residential habitats on Masig Island, Torres Strait, Queensland, Australia.* Asterisk* indicates species which are known to possess extra floral nectaries, confirmed off-site by an expert.* x* Species presence, *x*^Fl^ species with blooming flowers,* x*^Fr^ species with fruits.

## Data Availability

The datasets and R scripts supporting the conclusions of this article are available from the Research Data JCU platform at: https://doi.org/10.25903/NDEX-YQ83.

## Abbreviations

ATSBs: Attractive targeted sugar baits
GLM: Generalised linear model
GLMM: Generalised linear mixed-effects model
LMM: Linear mixed model
