# Supplementary material for: Sugar prevalence in Aedes albopictus differs by habitat, sex and time of day on Masig Island, Torres Strait, Australia
Source: Parasit Vectors. 2021 Oct 9;14:520. doi: 10.1186/s13071-021-05020-w (PMC8501651; doi:10.1186/s13071-021-05020-w)
Supplement: Supplementary file 1 — Additional file 1: Table S1. Coordinates for each station and distance to either the front or back door of the nearest inhabited dwelling. [file 13071_2021_5020_MOESM1_ESM.docx]

**Additional files**

**Additional file 1: Table S1** Coordinates for each station and distance to either the front or back door of nearest inhabited household.

| Station | X | Y | Distance to either the front or back door of nearest inhabited household (m) |
| --- | --- | --- | --- |
| Residential 1 | 143.4144 | -9.752181 | 10.9 |
| Residential 2 | 143.4121 | -9.751935 | 11.8 |
| Residential 3 | 143.4119 | -9.750992 | 12.1 |
| Residential 4 | 143.4122 | -9.750113 | 11 |
| Residential 5 | 143.4142 | -9.749846307 | 7.5 |
| Residential 6 | 143.4132 | -9.749425 | 13.7 |
| Residential 7 | 143.4147 | -9.750717011 | 8.8 |
| Residential 8 | 143.4167 | -9.750616 | 4.8 |
| Woodland 1 | 143.4096 | -9.751819204 | 101.5 |
| Woodland 2 | 143.4097 | -9.752930655 | 200.2 |
| Woodland 3 | 143.4109 | -9.752914624 | 87.6 |
| Woodland 4 | 143.411 | -9.751909095 | 53.1 |
| Woodland 5 | 143.4096 | -9.750385754 | 54.1 |
| Woodland 6 | 143.4091 | -9.749635192 | 155.6 |
| Woodland 7 | 143.4081 | -9.749246741 | 254.2 |
| Woodland 8 | 143.4105 | -9.750851064 | 48.9 |
