# Supplementary material for: Sugar prevalence in Aedes albopictus differs by habitat, sex and time of day on Masig Island, Torres Strait, Australia
Source: Parasit Vectors. 2021 Oct 9;14:520. doi: 10.1186/s13071-021-05020-w (PMC8501651; doi:10.1186/s13071-021-05020-w)
Supplement: Supplementary file 2 — Additional file 2: Table S2. Identified plant species at stations in woodland and residential habitats on Masig Island, Torres Strait, Queensland, Australia. Asterisk indicates species which are known to possess extra floral nectaries, confirmed off-site by an expert. x Species presence, xFl species with blooming flowers, xFr species with fruits. [file 13071_2021_5020_MOESM2_ESM.docx]

**Additional file 2: Table S2** Identified plant species at stations in woodland and residential habitats on Masig Island, Torres Strait, Queensland, Australia. “x” denotes species presence, “x^Fl^” denotes species with blooming flowers, “x^Fr^” denotes species with fruits. *** indicates species which are known to possess extra floral nectaries confirmed off site by expert opinion.

|  |  | Habitat type | | | | | | | | | | | | | | | |
| --- | --- | --- | --- | --- | --- | --- | --- | --- | --- | --- | --- | --- | --- | --- | --- | --- | --- |
| Plant type |  | Residential | | | | | | | | Woodland | | | | | | | |
|  | Stations | 1 | 2 | 3 | 4 | 5 | 6 | 7 | 8 | 1 | 2 | 3 | 4 | 5 | 6 | 7 | 8 |
|  | Species (Family) |  | | | | | | | | | | | | | | | |
| Woody plants | *Archontophoenix alexandrae* (Arecaceae*)* | x | x | x |  |  |  | x |  |  |  |  |  |  |  |  |  |
|  | *Casuarina equisetifolia* (Casuarinaceae) |  |  |  |  |  |  | x |  | x |  | x | x | x | x | x | x |
|  | *Guettarda speciosa* (Rubiaceae) |  |  |  |  |  |  |  |  |  |  |  | x^Fl^ | x^Fl,Fr^ |  |  | x |
|  | *Drypetes deplanchei* (Putranjivaceae) |  |  |  |  |  |  |  |  |  | x^Fr^ | x^Fr^ | x^Fr^ |  | x^Fr^ | x^Fr^ | x^Fr^ |
|  | *Eugenia reinwardtiana* (Myrtaceae) |  |  |  |  | x^Fr^ |  | x |  |  |  |  |  |  |  |  |  |
|  | *Gymnosporia inermis* (Celastraceae) |  |  |  |  |  |  |  |  |  | x^Fr^ |  |  |  |  |  |  |
|  | *Pandanus tectorius* (Pandanaceae) |  |  |  | x^Fr^ |  |  |  |  |  |  |  |  |  |  |  |  |
|  | *Planchonella obovate* (Lecythidaceae) |  |  |  |  |  |  |  |  |  |  |  |  |  |  | x |  |
|  | *Premna serratifolia* (Lamiaceae) |  | x^Fl^ |  |  |  |  |  |  | x |  |  |  |  |  |  |  |
|  | *Terminala catappa* (Combretaceae) | x |  |  |  |  | x^Fl^ | x |  |  | x^Fl^ | x^Fr^ |  |  |  |  |  |
| Herbaceous plants | *Tamarindus indica** (Fabaceae) |  |  | x^Fr^ |  |  |  |  |  |  |  |  |  |  |  |  |  |
|  | *Clitoria ternatea* (Fabaceae) |  | x^Fl^ |  |  |  |  |  |  |  |  |  |  |  |  |  |  |
|  | *Boerhavia sp.* (Nyctaginaceae) |  |  |  |  |  |  | x^Fl^ |  |  |  |  |  |  |  |  |  |
|  | *Catharanthus roseus* (Apocynaceae) | x^Fl^ | x^Fl^ | x^Fl^ | x^Fl^ | x^Fl^ | x^Fl^ | x^Fl^ |  |  |  |  |  |  |  |  |  |
|  | *Commelina cyanea* (Commelinaceae) |  | x^Fl^ |  | x^Fl^ |  |  | x^Fl^ |  |  |  |  |  |  |  |  |  |
|  | *Crinum asiaticum* (Amaryllidaceae) |  |  |  |  |  |  |  | x^Fl^ |  |  |  |  |  |  |  |  |
|  | *Euphorbia cyathophora** (Euphorbiaceae) |  |  | x^Fl^ |  |  |  |  | x^Fl^ |  |  |  |  |  |  |  |  |
|  | *Euphorbia heterophylla** (Euphorbiaceae) |  |  | x |  | x^Fl^ | x^Fl^ |  | x^Fl^ |  |  |  |  |  |  |  |  |
|  | *Galactia tenuiflora* (Fabaceae) |  |  |  | x^Fl^ |  |  |  |  |  |  |  |  |  |  |  |  |
|  | *Hibiscus rosa-sinensis** (Malvaceae) | x^Fl^ |  |  | x^Fl^ | x^Fl^ |  | x^Fl^ | x^Fl^ |  |  |  |  |  |  |  |  |
|  | *Ipomoea pes-caprae** (Convolvulaceae) |  |  |  |  |  | x^Fl^ |  |  |  |  |  |  |  |  |  |  |
|  | *Ixora coccinea** (Rubiaceae) | x^Fl^ |  |  | x^Fl^ | x^Fl^ |  | x^Fr^ |  |  |  |  |  |  |  |  |  |
|  | *Lantana camara* (Verbenaceae) |  | x^Fl^ |  |  |  |  |  |  |  |  |  |  |  |  |  |  |
|  | *Ruellia tuberosa** (Acanthaceae) | x^Fl^ |  |  |  |  |  |  |  |  |  |  |  |  |  |  |  |
|  | *Sida acuta* (Malvaceae) |  |  |  |  | x^Fl^ | x^Fl^ |  |  |  |  |  |  |  |  |  |  |
|  | *Stachytarpheta jamaicensis** (Verbenaceae) |  | x^Fl^ | x^Fl^ |  | x^Fl^ | x^Fl^ |  |  |  |  |  |  |  |  |  |  |
|  | *Stylosanthes humilis* (Fabaceae) |  | x^Fl^ | x^Fl^ |  | x^Fl^ |  |  |  |  |  |  |  |  |  |  |  |
|  | *Tacca leontopetaloides** (Taccaceae) |  |  |  |  |  |  | x^Fl^ |  |  |  |  |  |  |  |  |  |
|  | *Tridax procumbens* (Asteraceae) | x^Fl^ | x^Fl^ | x^Fl^ | x^Fl^ | x^Fl^ | x^Fl^ | x^Fl^ | x^Fl^ |  |  |  |  |  |  |  |  |
|  | *Turnera ulmifolia** (Passifloraceae) |  |  |  | x^Fl^ |  |  | x^Fl^ |  |  |  |  |  |  |  |  |  |
|  | **Species richness** | **7** | **9** | **8** | **8** | **9** | **7** | **12** | **5** | **2** | **3** | **3** | **3** | **2** | **2** | **3** | **3** |
